# Equine Trypanosomiasis: Molecular Detection, Hematological, and Oxidative Stress Profiling

**DOI:** 10.1155/2024/6550276

**Published:** 2024-08-16

**Authors:** Mostafa Shoraba, Salma A. Shoulah, Faysal Arnaout, Abdelfattah Selim

**Affiliations:** Department of Animal Medicine (Infectious Diseases) Faculty of Veterinary Medicine Benha University, Toukh 13736, Egypt

## Abstract

Surra caused by *Trypanosoma evansi* (*T. evansi*) is widely distributed and has significant impact on equine sector and international trades. However, there are no available data about the genetic characterization of this parasite in horses in Egypt. So, the goal of this study was to study the molecular characterization of *T. evansi* in horses and determine the changes in hematological parameters and oxidative stress associated with *T. evansi* infection. A total of 12 horses were examined using PCR targeting RoTat 1.2 VSG gene, and we evaluated the changes in hematological and oxidative stress between infected and healthy animals. The results revealed a notable reduction in red blood cell (RBC), hematocrit (HCT), and hemoglobin (Hb) levels in the infected horses, as compared to the control healthy group. In contrast, the infected group showed a substantial increase in mean corpuscular hemoglobin concentration (MCHC), mean corpuscular hemoglobin (MCH), and mean corpuscular volume (MCV). In addition, the infected group exhibited monocytopenia, eosinopenia, and notable lymphocytopenia. Regarding oxidative stress profile, the infected horses showed decreased levels of glutathione (GSH), superoxide dismutase (SOD), catalase (CAT), and antioxidant capacity (TAC) compared to the control group. Moreover, the PCR assay targeting RoTat 1.2 VSG gene revealed positive specific band (205 bp) in all examined samples for *T. evansi* and the phylogenetic analysis demonstrated that strain of this study is closely related to *T. evansi* isolate in horses from India (MT501210) while showed difference from sequences of other species. The results emphasize the changes in blood composition and the body's response to oxidative stress caused by *T. evansi* infection in horses.

## 1. Introduction

Many diseases of major importance in equine go undiagnosed and unreported [1–5]. Trypanosomiases, caused by unicellular flagellate protozoa from the genus *Trypanosoma,* are among these diseases. *T. evansi* and *T. vivax*, often found in Africa, the Middle East, Asia, and Latin America, are the most clinically significant species that can infect horses [6]. *T. evansi* is mostly transmitted mechanically by hematophagous insects from the *Tabanidae* and *Stomoxidae* families; however, ticks have also been transmitting the disease [7].

Clinical signs can vary depending on the animal's immune system and disease stage. They can include progressive weight loss despite an insatiable appetite, intermittent fever, weakness, anemia, pale mucous membranes, limb and ventral edema, urticaria plaques, keratitis and conjunctivitis, and paralysis of hindquarters and lips. If animal left untreated, these symptoms can lead to death [8, 9]. Moreover, the infection with *T. evansi* is commonly associated with reductions in hemoglobin (Hb), packed cell volume (PCV), and red blood cell count (RBC) [10, 11].

*T. evansi* has been reported in many geographical locations in Egypt, affecting buffalo, cattle, donkeys, camels, sheep, and goats [12, 13]. Unfortunately, there is a lack of data regarding the occurrence of *T. evansi* in horses in Egypt [14].

*Trypanosome* infection produces large amounts of reactive oxygen species (ROS) and free radicals, which function as cytotoxic agent [15]. Accumulation of ROS within cells has the ability to damage biomolecules, with lipids being particularly vulnerable unless regulated by an efficient antioxidant scavenging mechanism [16].

Antioxidant enzymes have a variety of defence mechanisms that successfully regulate reactive oxygen species (ROS) levels to ensure they remain in sufficient quantities. Two antioxidant enzymes, SOD and CAT, work along with nonenzymatic antioxidants such as GSH to remove free radicals and reduce oxidative damage to cells. Even minor differences in the amounts of these antioxidant enzymes can have a major impact on the ability of cellular lipids, proteins, and DNA to resist oxidative damage [17]. In experimental horses infected with *T. evansi*, blood levels of catalase, superoxide dismutase, and glutathione showed significant decreases [18].

Samples of blood, lymph, milk, cerebrospinal fluid, and preputial or vaginal discharges are used for detection of *T. evansi* [7]. Numerous tests can be employed, such as the inoculation of laboratory animals as mice, blood smears, molecular detection using polymerase chain reaction (PCR), and serological analysis like the complement fixation test, indirect immunofluorescence antibody test (IFAT), enzyme-linked immunosorbent assay (ELISA), and card agglutination test for trypanosomiasis (CATT) [19].

Molecular approaches are increasingly becoming more important as the preferred technique to detect pathogens [20]. DNA-based technologies, such as polymerase chain reaction (PCR), have been widely employed for diagnosing trypanosomiasis infection in a variety of animal species, including camels, horses, cattle, and pets. This approach is extremely sensitive and specific, capable of detecting all phases of parasite infection [21]. Several target sequences, including internal transcribed spacer region (ITS), ribosomal DNA, VSG genes, and kinetoplast DNA, have been identified as dependable targets for detecting *T. evansi* [21].

*Trypanosomes* have the unique capacity to switch the class of Variant Surface Glycoproteins (VSGs), which allows the parasite to survive by dodging the host's immune response. Among these, RoTat 1.2 VSG emerges as the primary variable antigen type (VAT), expressed during the early, middle, and late stages of *T. evansi* infection [22]. Consequently, RoTat 1.2 VSG serves as a validated molecule for both serological [23] and molecular detection [24] of *T. evansi* infection.

This study aimed to evaluate the alteration in hematobiochemical parameters and oxidative stress profile associated with *T. evansi* infection. In addition, genetic identification and phylogenetic analysis were performed for *T. evansi* isolate from horses based on RoTat 1.2 VSG gene.

## 2. Materials and Methods

### 2.1. Ethical Statement

The study design received approval from the Research Ethical Committee of Faculty of Veterinary Medicine, Benha University, Egypt (ethical number: BUFVTM39-09-23). All procedures of the study were carried out in accordance with regulation of ethical committee of Faculty of Veterinary Medicine, Benha University.

### 2.2. Animals and Sampling

**Figure 1:**
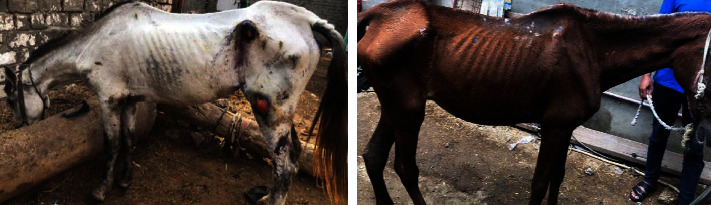
The diseases horses showed severe emaciation and weakness.

A total of 12 horses were subjected for examination, and seven of them (infected group) showed clinical signs of trypanosomiasis like emaciation, pale mucous membrane, and difficulty in walking as shown in Figure 1, while the rest of the five horses were apparently healthy. Blood samples (5 mL) were collected aseptically from jugular vein using vacutainer tubes have EDTA which used for hematological and molecular examination. In addition, another samples was collected in clean tube without anticoagulant to separate the serum for biochemical analysis.

### 2.3. Hematological Parameters

An automated cell counter (Celltac Alpha, Nihon Kohden Europe GmbH, Rosbach, Germany) was employed to assess various blood parameters, including red blood cells (RBC), mean corpuscular volume (MCV), hemoglobin concentration (HGB), hematocrit (HCT), mean corpuscular hemoglobin concentration (MCHC), mean corpuscular hemoglobin (MCH), white blood cell (WBC), monocytes (MO), lymphocytes (LY), and eosinophils (EO).

### 2.4. Oxidative Stress Evaluation

The levels of MAD, SOD, CAT, TAC, and GSH were measured in serum samples using commercial kits (Biodiagnostic, Giza, Egypt) according to the guidelines of the manufacturer.

### 2.5. DNA Extraction and PCR Assay

The DNA was extracted from the prepared blood sample according to the manufacturer's instructions using the QIAamp DNA Kit (Qiagen, Hilden, Germany). The extracted DNA was kept at −20° until use. Amplification of RoTat 1.2 VSG gene for detection of *T. evansi* was performed using the specific pair of primers RoTat-F:5′-GCGGGGTGTTTAAAGCAATA-3′ and RoTat-R:5- ATTAGTGCTGCGTGTGTTCG-3′ for detection of 205 bp product size [25]. The PCR assay was performed in 25 *μ*l volumes containing 5 *µ*l DNA template, 1 *μ*l of each primer (20 pmol/*μ*l), 12.5 *μ*l of emerald Amp GT PCR Master mix (2xprimer), and 5.5 *μ*l PCR grade water under the following condition: initial denaturation at 94°C for 5 min followed by 35 cycles of denaturation at 94°C for 30 sec, annealing at 52°C for 30 sec, and extension at 72°C for 40 sec. After the last the cycle, the mixture was incubated at 72°C for 10 min. The positive control for *T. evansi* was provided from Animal health Research Center, Giza, Cairo. The amplification products were analyzed by electrophoresis on 1.5% agarose gel.

### 2.6. Sequencing and Phylogenetic Analysis

The purified PCR products of a highly concentrated DNA samples was used for sequencing using the sample primers of PCR assay. The automated DNA sequencer Applied Biosystems 3130 was used to sequence the DNA (ABI, 3130, USA), employing the BigDye Terminator V3.1 cycle sequencing kit (Perkin-Elmer/Applied Biosystems, Foster City, CA). This was followed by an initial BLAST® analysis to determine sequence identity with GenBank database [26].

The sequences were assembled and edited using the BioEdit program before being entered into GenBank with an accession number (LC789026). The partial RoTat 1.2 VSG gene sequences were aligned with previously reported gene sequences in the GenBank database (https://www.clustalw.genome.jp) using CLUSTAL W [27]. The neighbor-joining tree approach was performed with 500 bootstrap replicates to generate a phylogenetic tree based on the Kimura 2-parameter model for nucleotide sequences using MEGA7 [28].

## 3. Results

### 3.1. Hematological Parameter Findings

In the infected horses, there was a notable decrease in the mean values of RBCs and HCT, whereas Hb exhibited a statistically significant reduction (*p*=0.021) compared to the healthy group. Conversely, a significant increase (*p* < 0.05) in the mean values of MCHC, MCH, and MCV was observed in the infected animals relative to the control healthy group. Additionally, the infected animals exhibited monocytopenia, eosinopenia, and significant lymphocytopenia (*p*=0.022) when compared to the control healthy group as shown in Table 1.

### 3.2. Oxidative Stress Parameters

In the infected horses, a reduction in the levels of GSH, SOD, CAT, and TAC was observed compared to the control group. Conversely, there was a significant (*p* < 0.0001) increase in the level of MDA in the infected animals relative to the control healthy group as shown in Table 2.

### 3.3. PCR Amplification and Phylogenetic Analysis

**Figure 2:**
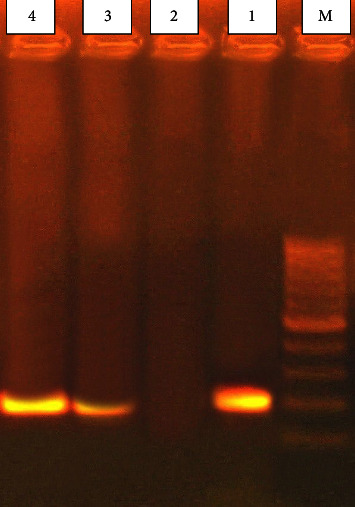
The infected animals showed detected specific band (205 bp) for *T. evansi*. M: molecular marker (1000 bp ladder); lane 1: positive control; lane 2: negative control; lane 3 and 4 are natural infected samples.

**Figure 3:**
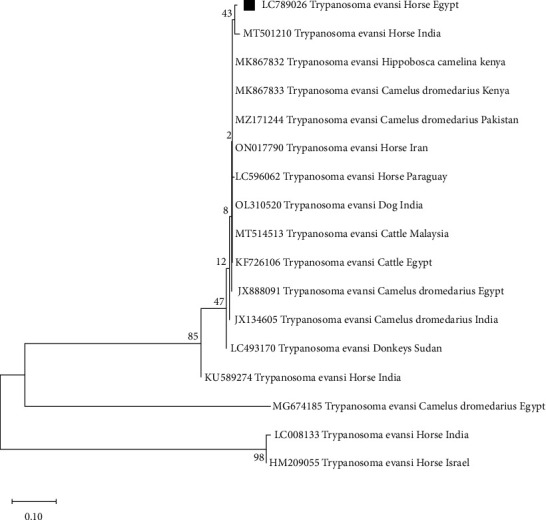
Phylogenetic analysis for Egyptian *T. evansi* based on RoTat 1.2 VSG gene with other *T. evansi* sequences from different species and countries in GenBank. The neighbor-joining method was employed to create the unrooted phylogenetic tree. Bootstrap values were calculated as percentages at each node using 500 replicates. The sequence of present study is highlighted with black square.

The considered positive samples for *T. evansi* showed single specific band of 205 bp size upon agarose gel electrophoresis, Figure 2. The *T. evansi* sequence of this study was deposited in the NCBI database, receiving the accession number LC789026. The phylogenetic analysis revealed that the sequence of *T. evansi* of this study was seen to be located in the same clade with the *T. evansi* isolate from horses from India (MT501210) while it is genetically distinct from other *T. evansi* sequences of other species as MK867833 from camels from Kenya, OL310520 from dogs from India, KF726106 from cattle from Egypt, and other strains from horses from other countries like India and Israel as shown in Figure 3.

## 4. Discussion

The hematological parameter findings in the infected animals revealed significant alterations in various blood components compared to the control group. There was a reduction in RBC, HCT, and Hb levels in infected horses, which indicated the presence of anemia as previously reported in trypanosomiasis-positive equines [10] and camels [29, 30]. Anemia is thought to be a significant and crucial sign of an animal's trypanosomiasis infection.

The microtubule-reinforced bodies and forceful flagella lashing activity of the million-strong organisms induce mechanical injury to erythrocytes, which in turn causes the complex pathophysiology of anemia in trypanosomiasis [31]. MCV, MCH, and MCHC are red cell indices that are used to identify the type of anemia [32].

In our study, the infected horses exhibited macrocytic anemia characterized by elevated MCHC, MCH, and MCV values, and this finding was in contrast to those reported by Kagira et al. [33] who observed a sharp decrease in MCV and a little reduction in MCH and MCHC which were the hallmarks of the early stages of microcytic hypochromic type anemia. These differences in the results may be attributed to stage of disease progression and underlying pathophysiological mechanisms [34].

Also, the elevated MCV may be the only indicator of conditions like vitamin B12 or folate deficiency and anemia is caused by parasitic infections, which interfere with absorbent surfaces, physically obstruct the intestinal lumen, produce proteolytic substances, and consume nutrients intended for the body [35]. This may be the underlying reason of our observations, as the increasing anemia exhibited in this case is known as anemia of chronic disease or inflammation and is typically found in susceptible animals [36]. It is characterized by heightened erythrophagocytosis and poor erythropoiesis due to altered iron hemostasis and persistent secretion of proinflammatory cytokines such as IFN-*γ*, TNF, IL-1, and IL-6 [37] and that could alter red cell indices, leading to increased MCV, MCH, and MCHC values.

The present findings showed decrease in eosinophils, and these corroborated with a previous study that demonstrated a reduction in horses infected by *T. evansi* [34]. This finding might be explained by the fact that helminth migration through host tissues plays a crucial part in inducing increases in tissue eosinophilic inflammation and blood eosinophils, and helminths that stay inside the intestinal lumen may not cause an eosinophil response [38].

Interestingly, this study revealed a significant decrease in lymphocytes in infected horses as previously reported by Pal et al. [34]; this could be related to several infectious pathogens that may produce a drop in lymphocyte numbers due to inflammation. These reductions are probably caused by enhanced lymphocyte migration to lymphoid tissues, increased margination and emigration of lymphocytes to the site of inflammation, and decreased lymphocyte efflux out of lymphoid tissues [39].

One of the main processes in the pathophysiology of trypanosomiasis is oxidative stress [40]; this is due to the host's decreased RBC capacity for antioxidants [41]. Reductions in the levels of GSH, SOD, CAT, and TAC in the infected animals demonstrate a decrease in antioxidant defence mechanisms, while a significant elevation in MDA levels in the infected animals indicates enhanced lipid peroxidation and oxidative damage. These findings are consistent with earlier research by Ranjithkumar et al. [11] and Saleh et al. [42]. This might be explained by the fact that the *T. evansi* infection releases sialidase and phospholipase, which cause damage to the erythrocyte membrane and the development of insulted red blood cells (RBCs). These RBCs then produce reactive oxygen species (ROS), which increase lipid peroxidation in RBCs and cause oxidative stress [43]. These findings suggest that trypanosomosis leads to severe protein oxidation, lipid peroxidation, and reduction in the antioxidant enzyme activity [15].

The RoTat 1.2 gene is expressed by most *T. evansi* strains. The RoTat 1.2-based PCR assay is highly sensitive and specific, making it effective for diagnosing trypanosomosis in different animal species [44, 45]. This understanding is essential for refining diagnostic procedures, developing effective control strategies, and treating *T. evansi* infections.

In the present study, the PCR assay targeting RoTat 1.2 VSG gene was used for detection of *T. evansi* in horses because the primers targeting this gene are more sensitive and specific for pathogen and help in early detection, identification of positive host, and consequently early effective treatment [46, 47]. These results come in accordance with findings of previous studies used the similar part of the RoTat 1.2 VSG gene in PCR assays to detect *T. evansi* [48].

Moreover, this *T. evansi* isolate clustered within the same clade with previously *T. evansi* isolate from India (MT501210), while exhibiting divergence from other sequences, including those from Egypt (MG674185), India (LC008133), and Israel (HM209055). Another noteworthy finding in this study distinct relationship between *T. evansi* isolate and other *T. evansi* isolates from other species from different countries like Egypt, India, and other countries. *T. evansi* isolate of this is clear divergence from isolates of Egypt and India of the same host species (Equine). It could be attributed to the diversity of the RoTat 1.2 VSG gene in Egyptian trypanosome isolates from horses associated to long-term parasite persistence due to chronic nature of the disease.

Our findings align with previous phylogenetic studies on *T. evansi* using the RoTat 1.2 VSG gene [49], which demonstrated genetic diversity and variability among populations in various places. The complicated evolutionary history for *T. evansi* may be attributed to the movement of diseased animals and insect vectors, leading to repeated hybridization occurrences between different strains [50].

Additionally, it may be linked to non-RoTat 1.2 VSG *T. evansi*, a variant described from Africa [5, 51–54]. Even though this is just a preliminary finding, it is undeniable that RoTat 1.2 VSG is selectively present in isolates from various hosts [55]. The present study has some limitations as few number of horse were examined, sequencing was performed for one sample and absent of blood smear diagnosis for the examined animals.

## 5. Conclusions

Oxidative damage to erythrocytes may contribute to anemia in horses infected with *T. evansi.* This can be used as a marker for both latent and recent infections when correlated with other hematobiochemical indicators. PCR can quickly and reliably detect *T. evansi* infection in horses, particularly when parasitemia is low. Using PCR in the field would not only help diagnose and treat individual animals but also eliminate the reservoir of infection, reducing the threat to equine and camel herds where blood sucking mechanical vectors are present. Moreover, *T. evansi* is circulating in Egyptian horses, thus regular monitoring at is recommended to implement and effective control measure. Further studies are needed to detect other *Trypanosoma* spp. and to study the alteration of hematological profile in case of coinfection.

## Figures and Tables

**Table 1:** Hematological parameters (mean ± SE) of infected and noninfected equine by *T. evansi*.

Parameters	Control	Infected	*p* value
RBCs	11.07 ± 2.38	8.03 ± 1.21	0.146
WBC	5.69 ± 0.54	10.28 ± 0.13	0.021^∗^
HGB	9.96 ± 0.32	4.57 ± 0.32	0.021^∗^
HCT	29.48 ± 1.39	21.47 ± 4.9	0.142
MCV	44.17 ± 0.66	59.23 ± 2.55	0.02^∗^
MCH	11.76 ± 0.47	28.4 ± 3.8	0.04^∗^
MCHC	22.46 ± 0.38	32.8 ± 0.1	0.002^∗^
LY	5.26 ± 0.27	2.43 ± 0.37	0.022^∗^
MO	0.63 ± 0.09	0.36 ± 0.03	0.156
EO	0.26 ± 0.14	0.07 ± 0.06	0.321

^∗^The result is considered significant if *p* value <0.05.

**Table 2:** Oxidative stress markers (mean ± SE) in infected and noninfected equine by *T. evansi*.

Animal	MDA	GSH	SOD	CAT	TAC
	(nmol/mL)	(U/mL)	(U/mL)	(mM/L)	
Infected	75.25 ± 0.31	2.47 ± 0.30	271.90 ± 0.29	10.50 ± 0.33	0.33 ± 0.23
Control	16.85 ± 1.08	3.27 ± 1.34	291.29 ± 1.97	17.58 ± 1.11	1.01 ± 0.23
*p* value	<0.0001^∗^	0.676	0.481	0.013	0.272

^∗^The result is considered significant if *p* value <0.05.

## Data Availability

The datasets used and analyzed during the current study are available from the corresponding author on reasonable request.
